# Assessment of Safety and Interference Issues of Radio Frequency Identification Devices in 0.3 Tesla Magnetic Resonance Imaging and Computed Tomography

**DOI:** 10.1155/2014/735762

**Published:** 2014-02-16

**Authors:** M. Periyasamy, R. Dhanasekaran

**Affiliations:** Syed Ammal Engineering College, Ramanathapuram, Tamil Nadu 623 502, India

## Abstract

The objective of this study was to evaluate two issues regarding magnetic resonance imaging (MRI) including device functionality and image artifacts for the presence of radio frequency identification devices (RFID) in association with 0.3 Tesla at 12.7 MHz MRI and computed tomography (CT) scanning. Fifteen samples of RFID tags with two different sizes (wristband and ID card types) were tested. The tags were exposed to several MR-imaging conditions during MRI examination and X-rays of CT scan. Throughout the test, the tags were oriented in three different directions (axial, coronal, and sagittal) relative to MRI system in order to cover all possible situations with respect to the patient undergoing MRI and CT scanning, wearing a RFID tag on wrist. We observed that the tags did not sustain physical damage with their functionality remaining unaffected even after MRI and CT scanning, and there was no alternation in previously stored data as well. In addition, no evidence of either signal loss or artifact was seen in the acquired MR and CT images. Therefore, we can conclude that the use of this passive RFID tag is safe for a patient undergoing MRI at 0.3 T/12.7 MHz and CT Scanning.

## 1. Introduction

In developed countries, it is believed that healthcare is one of the largest as well as fastest growing service industries [1] dealing with patient life and any shortfall in its quality of service has the potential to develop irrevocable and incurable loss to patients [2]. Medical errors, categorized as slips or lapses [3], are not only detrimental to patients' life but also incur further healthcare expenses [4, 5]. The medical error may occur at any point or at any stage in hospital care process. Some of them are incorrect patient identification [4–6], misinterpretation of hand written prescription [5], mislabeling of patient's bio samples [3], selection of wrong site for surgery [7], incorrect administration of drug [3, 5, 7–9], and improper transfusion of blood [3]. Among the aforementioned, incorrect patient identification is the most important issue to be addressed, and utmost care must be taken to ensure correct patient identification during the treatment process in hospitals. Patient safety can be enhanced by adopting suitable information technology (IT) tools in hospitals to minimize the occurrences of medical errors [5, 10].

Among several identification technologies available, RFID is termed as the emerging technology of the last decade [11] and it has enhanced the quality of process in retail marketing, supply chain management, inventory control, and logistics [12]. With the help of built in memory, each RFID tag can carry limited amount of information about the person or object to be tagged. RFID does not require line of sight for communication and it can read multiple numbers of tags simultaneously even in adverse light conditions [13]. By exploiting these characteristics, RFID can be employed in healthcare for numerous applications ranging from tracking and locating valuable assets [14], patient identification [6, 14], patient tracking, medication tracking, monitoring of doctors, and staff. With the introduction of RFID in healthcare, it is certain to improve the quality of medical services rendering [15] a decrease in the cost of healthcare [13], improvement in consistency and reliability in patient care [16] and an increase in nursing efficiency by reducing the burden on the nursing staff [5, 13].

In order to guarantee patient identification throughout the hospital stay, RFIDs must be attached to the wrists of the patients and these need not be removed till a patient leaves the hospital [6]. During a hospital stay, the patients with RFID tags mounted on their wrists may need to undergo magnetic resonance imaging (MRI) examination or computed tomography (CT) scan [6]. During these procedures, the RFID tags are exposed to harsh electromagnetic radiations from MRI [6, 7, 17, 18], as well as high intensity X-rays of CT scan [6]. However, patients carrying certain types of electronically activated implants, which are actually magnetically activated devices or electrical devices representing larger metallic masses and higher magnetic susceptibilities, are unacceptable for MR imaging procedure [19–24]. Such devices/implants in the presence of strong magnetic field interactions as well as the heating related to MR imaging process not only change the functionalities of the devices itself but may also produce MR imaging artifacts; consequently the presence of those devices/implants on patient affects the overall diagnostic use of MRI as well as the patients' safety. In few cases, certain type of devices was even found to cause severe injury to the patient [24]. Therefore, preexamination check by MRI operator for all the patients should be made mandatory to ensure that no patients carry contraindicated implants or devices to the MR imaging environment [25]. Similar guidelines are essential to characterise MRI issues for patients wearing RFID tags at 0.3 T MR imaging environment with regard to patient safety [6, 7, 17, 18]. According to American society for testing and materials (ASTM) standards [26, 27] and by [28], if the device does not pose risks to patient during MRI examination, it could be labeled as “MR safe” and if it is able to continue its operation after an MRI examination, then it is termed as “MR compatible.” Various combinations of factors including device size, orientation of the device, magnetic susceptibility of the device, and the pulse sequences performed determine the size of an artifact in MR images [29]. Depending on its severity, the artifacts are classified as no artifact, mild artifact, moderate artifact, and strong artifact [29]. As far as CT scan is concerned, although it could not harm the patient safety, it has the capacity to destroy the semiconductor structure of the RFIDs with its powerful X-rays [6].

The objective of our work was to evaluate the functional aspects and imaging artifacts for passive 13.56 MHz RFID tags, which are most suitable for medical applications [6, 8]. For this purpose we conducted in vivo testing at certain clinical conditions in 0.3 T/12.7 MHz MRI system and CT scan.

## 2. Related Literature

Lamberg [18] evaluated MRI issues for VeriChip human RFID implants at 1.5 Tesla MRI system and found that VeriChip implants are “MR Safe,” but not “MR Compatible” as the tested implant got deactivated after an MRI examination.

Cheng and Chai et al. [7] evaluated device functionality and data loss issues with regard to the use of the passive 915 MHz RFID tags at 1.5 T/64 MHz MRI system. After an MRI examination, RFID tags sustained no physical damage and the functionality of the RFID tags remained unaffected. There was no memory loss or data loss on those tested tags.

Titterington and Shellock [17] evaluated the MRI issues of an access port equipped with RFID tag operating at the frequency ranges of 129.0–133.2 KHz and 135.2–139.4 KHz at 1.5 T/64 MHz and 3.0 T/128 MHz MRI systems, respectively. The results indicated that the access port equipped with RFID tag is “MR Safe” as well as “MR Conditional” and it would be acceptable to use at both 1.5 T/64 MHz and 3.0 T/128 MHz MRI systems. However, it produces an artifact of considerable size in MR images when the imaging area of interest is the area where an access port is implanted.

Steffen et al. [6] examined the reliability and data integrity of passive 13.56 MHz RFID tags in 1.5 T, 3.0 T MRI systems, and in CT scan. They concluded that reliability and data integrity of RFID tags remain unaffected by electromagnetic radiations of MRI systems and X-rays of CT scan. However, the RFID tags mounted on the wrist of a patient introduced smaller artifacts in MR images of wrist. The size of an artifact is minimal and it did not impact the diagnostic quality. Steffen et al. [6] also pointed out that there is higher probability of passive 13.56 MHz RFID tags to exhibit change in functional aspects when they undergo MRI examination at lower magnetic field system such as 0.3 T or 0.5 T since its operating frequency is close to 13.56 MHz.

## 3. Materials

### 3.1. Radio Frequency Identification (RFID)

**Figure 1:**
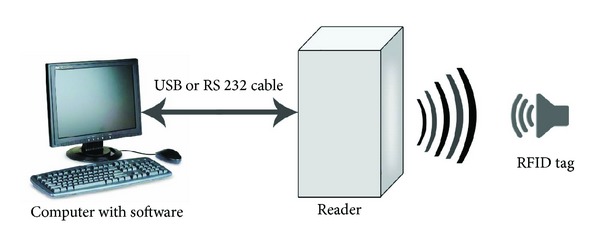
Block diagram of radio frequency identification (RFID) system.

A typical RFID system consists of an interrogator (also known as reader), tag, computer, and back-end software as shown in Figure 1. During operation, whenever a tag comes under the influence of the electromagnetic (EM) field of the reader, it transmits its unique identification (UID) number along with the identification (ID) of the reader. With the help of the software loaded in the computer connected to the RFID reader, one can perform read and write operations on tags. Depending on the power feed, tags are classified into active and passive. Active tags have built in battery and passive tags derive their power from electromagnetic field radiated by the reader. Active tags have the advantages of stable transmission capacity with relatively larger battery size [13] and better reading range with higher cost of tags and reader. Passive tags have the advantages of unlimited life with simple design [13] and no battery requirement. Single usage and cheaper cost involve in the implementation of such tags. Since the lower cost of the system and long term usage are the two important factors dominating the healthcare application, the passive RFID system becomes the appropriate choice for healthcare logistics process [13].

The RFID system used in this study is a Milfare CR500 Standard, passive, 13.56 MHz, compatible with heterogeneous standards such as ISO144A, ISO144B, and ISO15693. The antennas of all of the RFID tags are made up of copper or aluminium, and they do not contain weak ferromagnetic materials, a prerequisite for implant or medical device to undergo MRI testing [6]. Tables 1 and 2 present the technical specifications of the RFID reader and tags used in our study.

### 3.2. MRI System and CT Scan

Generally MRI is a safe imaging procedure used in clinical settings to create high quality images of internal organs of human body. It is also the most preferred diagnostic tool to get detailed analysis of anomalies or lesions present inside any organ. We evaluated the RFID for device functionality and image artifacts using standard MR imaging sequences at 0.3 T/12.7 MHz MRI system (AIRIS III, HITACHI, Japan). An MRI system with static magnetic field strength of 0.3 Tesla was selected as its operating frequency is close to the operating frequency of RFID tags used in this study. We used the CT scan machine made by General Electric, USA.

## 4. Methodology

We conducted this study with one human volunteer. Similar type of studies involving a human volunteer have also been reported in the literature earlier [6, 7]. Written consent was obtained, and the imaging procedure to be followed was also explained to the volunteer. This study was approved by local ethics committee of the hospital. Several studies [19–24] have already shown that certain implant (ossicular implants, CSF shunt valves and retinal prosthesis, etc.) or devices representing larger metallic masses and higher magnetic susceptibilities (stainless steel, titanium, and small magnets) do not cause hazards (no displacement, negligible heating effects, and no functional alterations) that may affect patients in an magnetic environment of 1.5 T and 3.0 T. The only concern is artifacts, which compromise the quality of MR images, especially when the size of an artifact is comparable to the size of the device. Size of the artifacts can be reduced by optimizing the MR imaging parameters such as change in pulse sequence from gradient-echo (GRE) to fast spin-echo (FSE) pulse sequence. The RFID tags used in this study have smaller metallic mass with less magnetic susceptibility and they create smaller diameter conducting loops compared to implants used [19]. Based on the above facts, we assumed that the MRI related issues including MRI heating and magnetic field interactions would be negligible for these RFID tags. At the same time, RFID tags and MRI scanner used for this study were working at close frequencies, consequently, tiny electronics of RFID tags, when placed at MRI environment, would absorb higher amount of energy due to resonance. However, this might cause the tags to get damaged as well as make changes in the functional aspects. Similar to the earlier studies [6, 7, 17, 18], these tags under MRI environment might also create artifacts in MR-images. Although patient safety is guaranteed with these tags, changes in functional aspects and image artifacts can impact the diagnostic use of MRI. This prompted us to undertake the current study, where we evaluated two issues related to MR imaging risks including functional alterations and imaging artifacts.

### 4.1. Functional Assessment at MRI

**Figure 2:**
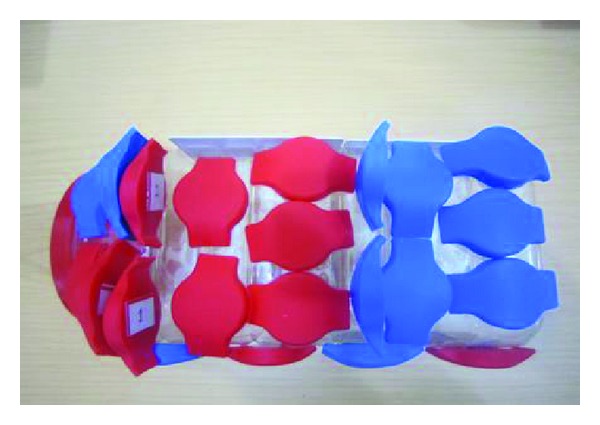
Group of RFID tags tested for this study.

In order to determine whether RFID tags could sustain physical damage or alterations in its function while in the environment of 0.3 T/12.7 MHz MRI system, fifteen samples of RFID tags with two different physical sizes (wrist band and ID card) were attached to the sides of a rectangular nonconductive plastic box of dimension of 24 cm × 13 cm × 10 cm (shown in Figure 2). In order to cover all possible situations that may arise while a patient with this RFID tag undergoes an MRI examination, we oriented these tags in three different directions: axial, coronal, and sagittal. All of the tags tested underwent functional evaluation before exposure to MR imaging conditions. The RFID reader was not included in the evaluation process and it was kept outside the MR system room. The plastic box containing RFID tags was positioned at the centre of the eight-channel wrist coil to ensure maximum exposure to all the tags. With the consented volunteer lying on the patient table and the plastic box containing RFID tags positioned at the centre of the wrist coil, an MRI was performed using four different pulse sequences representing typical techniques used for clinical MRI examination, running consecutively, for approximately two minutes per pulse sequence. The MRI sequences performed on each tag were listed in Table 3. A functional check was carried out on all RFID tags outside the MRI and CT scan area after they were scanned by MRI and CT scan. Correct function of the RFID tag was characterised by three factors in three steps after exposure to the CT and MRI examination: (i) UID of each RFID tag must be readable without any error [6], (ii) read and write operations should be carried out in all memory blocks of each tag without programmable error, and (iii) error free operation of the tag was assumed when the content that already stored in memory of each RFID tag was retrieved

### 4.2. Artifact Assessment at MRI

MRI artifacts were evaluated at 0.3 T/12.7 MHz MRI system by acquiring MR images of the volunteer with and without RFID tag attached to the wrist and placing it on wrist coil. We performed MRI examination using 0.3 T system with the following imaging sequences:

1. T_1_-weighted, spin echo pulse sequence, repetition time (300 ms), echo time (18 ms), section thickness (6 mm), field of view (26 cm), number of excitations (2), and matrix size (256 × 256);
2. T_2_-weighted, fast spin echo pulse sequence, repetition time (6100 ms), echo time (125 ms), section thickness (6 mm), field of view (26 cm), number of excitations (2), and matrix size (256 × 256).

For qualitative analysis of artifacts, we compared the MR images acquired with RFID tag on wrist with the MR images acquired without an RFID tag.

### 4.3. Functional Assessment at CT Scan

After MR imaging sequences, a CT examination was also performed using a standard procedure for abdominal examination in the presence of the RFID tags with a CT technique of 100 mAs at 120 kV. The exposure time for CT examination was 20 s.

## 5. Results 

After MRI and CT scanning, all of the fifteen tags tested exhibited proper function without any physical damage. We could read the UID of each tag with no indication of loss of memory or loss of data. In addition, read and write operations in all memory blocks of each tag remained functional. Previously saved content could be retrieved completely without alteration.

**Figure 3:**
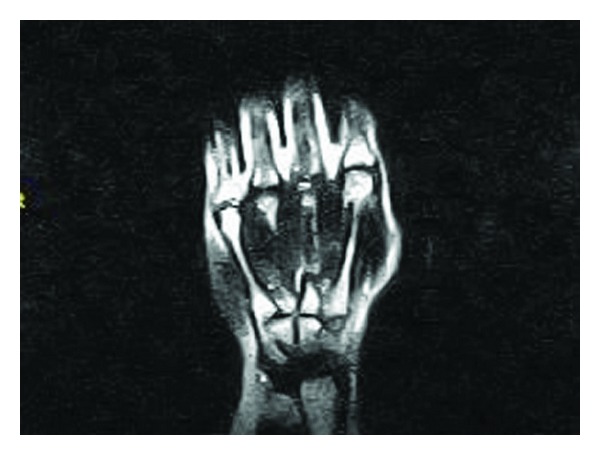
MR image of the volunteer without RFID tag attached to the wrist (Imaging Sequence: T_1_—Spin echo Coronal).

**Figure 4:**
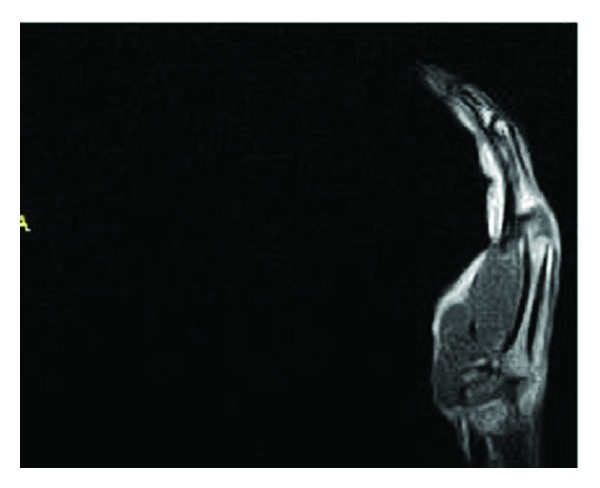
MR image of the volunteer without RFID tag attached to the wrist (imaging sequence: T_2_—Fast spin echo Sagittal).

**Figure 5:**
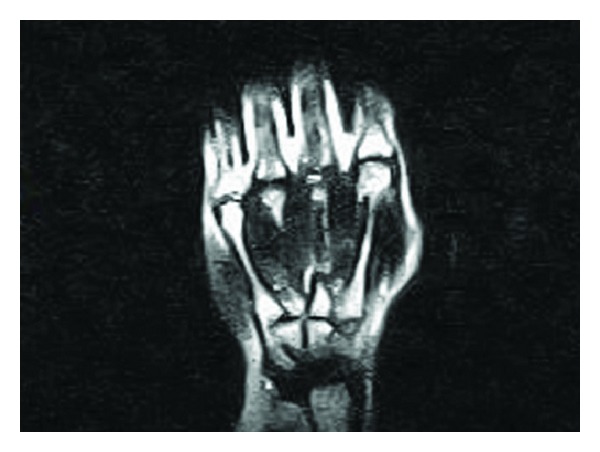
MR image of the volunteer with RFID tag attached to the wrist (imaging sequence: T_1_—Spin echo Coronal).

**Figure 6:**
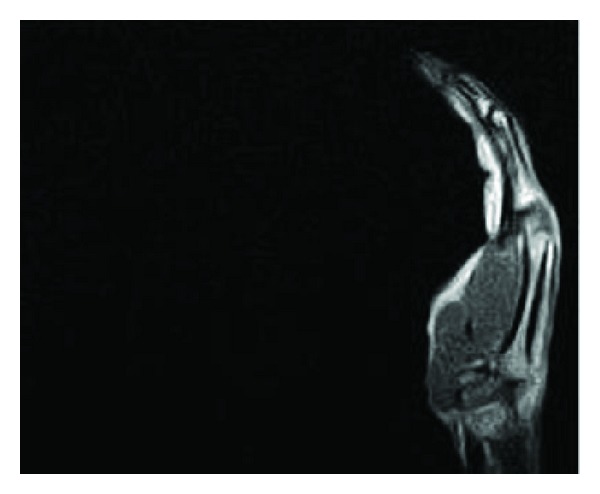
MR image of the volunteer without RFID tag attached to the wrist (imaging sequence: T_2_—Fast spin echo Sagittal).

No artifacts or signal loss were found in MR images of the volunteer wearing the RFID tags on the wrist, positioned near to the skin surface. Also the quality of the images was not impaired due to the presence of RFID tags on wrist of the volunteer. The MR images of the volunteer without and with RFID tags attached to the wrist are shown Figures 3, 4, 5, and 6. No artifacts were found even in CT images, and the presence of RFID tags did not influence the quality of images captured.

## 6. Discussion

We performed this study to evaluate device safety as well as image artifact issues related to use of passive 13.56 MHz RFID tags in 0.3 T/12.7 MHz MRI system and CT scan at certain clinical conditions. We successfully demonstrated that the RFID tags used in this study did not sustain physical damage after being subjected to several MR imaging sequences and CT scanning. Importantly, these tags also showed the capacity to retain their full functionality even after being exposed to harsh electromagnetic environments coupled with MRI system and harmful X-rays of CT scan. After MRI and CT scanning, RFID tags lose neither any data nor was any alteration in its memory. This was also evident from earlier studies [6, 7, 17] involving characterization of RFID tags operating at different frequency bands in 1.5 T or 3.0 T MRI systems except a study undertaken by Lamberg [18]. On the other hand, Steffen et al. [6] predicted that low field MRI systems with 0. 3 T or 0.5 T may likely induce more interference to passive 13.56 MHz RFID tags since its operating frequency is close to 13.56 MHz. However, our results confirmed occurrences of no such interference as well as no harmful effects to the volunteer. Patients wearing these RFID tags on their wrists may not need to take off while undergoing MRI and CT examination.

Although we did not specifically concentrate on the issues such as device heating or device movement, the volunteer felt no heating on the surface of the skin where these RFID tags were worn and no device movement was observed. Interestingly, the absence of artifacts was quite surprising and encouraging. This could be due to the fact that the antennas of the RFID tags were made of either aluminum or copper having lesser magnetic susceptibility compared to titanium or stainless steel. Also the MRI scanner involved in this study was of low field, 0.3 T. The artifacts have been reported to appear in MR images involving higher field systems including 1.5 T and 3.0 T [6, 7, 17, 18]. Also the size of the artifact depends on several factors; however, in most cases they do not obstruct the diagnostic quality. We did not even find any alteration in the CT images when the volunteer underwent scanning wearing these RFID tags on the wrist. With regard to device functionality and image artifact, we found that passive 13.56 MHz RFID tags are safe to use at 0.3 T/12.7 MHz MRI system and CT scan.

There are some limitations in our study. The whole study was performed for a single frequency in both 0.3 T /12.7 MHz MRI system and CT scan by a single radiologist. Although passive RFID tags are available in several frequency bands, we limited ourselves to use of passive 13.56 MHz RFID tags supplied by Switzer Instruments, Chennai, India. Extensive research is required to explore the possibility of using RFID tags at other frequency bands under 0.3 T/12.7 MHz MRI and CT scan. The reason for not using active RFID tags was simply because these tags are more costly compared to the passive tags, and hospitals require much cheaper systems to be installed [6]. Although there are few limitations in our study, it confirmed the advantages of using passive 13.56 MHz RFID tags on patient safely in 0.3 T/12.7 MHz MRI system and CT scan resulting in no artifacts in images as well as no alteration in the functionality of tag itself.

## 7. Conclusions

Based on our research findings, it is evident that passive 13.56 MHz RFID tags worn on patient's wrist are safe in 0.3 T/12.7 MHz MRI system as well as in CT scan and also these tags need not be removed during MRI and CT examination. Thus, it guarantees the patient identification throughout the entire procedure with no harmful effects either on device functionality or patient safety or quality of MRI and CT images acquired. Our results are specific to passive 13.56 MHz RFID tags tested under MRI and CT scan, especially while performing various MR imaging sequences and CT scanning protocol in 0.3 T/12.7 MHz MRI system and CT scan, respectively.

## Figures and Tables

**Table 1:** Technical specifications of RFID reader used in our study.

Parameter	Specifications
Operating frequency	13.56 MHz
Multitag read capability	No
Communication standard	USB
Immunity to noise and interference	Yes
Read/write distance	Up to 60 mm
Operating temperature	−20°C to 50°C
Transmission speed	19200 bps
Output power	0.75 mW

**Table 2:** Technical specifications of RFID tag used in our study.

Parameter	Specifications
Tag type	Passive, wrist band and ID card type
Operating frequency	13.56 MHz
UID (unique identification number)	4 Bytes
Memory	1 KB

**Table 3:** MRI sequences performed on each tag at 0.3 T/12.7 MHz for testing device functionality.

Pulse sequence	T_1_-SE	T_2_-SE	T_1_-FSE	T_2_-FSE
Image conditions				
*T*_*R*_ (ms)	300	600	4430	6100
*T*_*E*_ (ms)	18	250	120	125
Flip angle	90°	90°	90°	90°
Field of view (cm)	26	26	26	26
Section thickness (mm)	6	6	6	6
Imaging plane	Sagittal	Axial	Sagittal	Coronal

T_1_-SE: T_1_-weighted spin echo; T_2_-SE: T_2_-weighted spin echo; T_1_-FSE: T_1_-weighted fast spin echo; T_2_-FSE: T_2_ weighted fast spin echo; *T*_*R*_: recovery time; *T*_*E*_: echo time.
